# Metabolomic analysis of longissimus from underperforming piglets relative to piglets with normal preweaning growth

**DOI:** 10.1186/s40104-018-0251-3

**Published:** 2018-04-26

**Authors:** Timothy G. Ramsay, Margo J. Stoll, Amy E. Shannon, Le Ann Blomberg

**Affiliations:** Animal Biosciences and Biotechnology Laboratory, U.S. Department of Agriculture, Agricultural Research Service, Beltsville, MD 20705 USA

**Keywords:** Growth rate, Longissimus, Metabolome, Neonate, Swine

## Abstract

**Background:**

Recent increases in intra-litter variability in weaning weight have raised swine production costs. A contributor to this variability is the normal birth weight pig that grows at a slower rate than littermates of similar birth weight. The goal of this study was to interrogate biochemical profiles manifested in skeletal muscle originating from slow growing (SG) and faster growing littermates (control), with the aim of identifying differences in metabolic pathway utilization between skeletal muscle of the SG pig relative to its littermates. Samples of longissimus muscle from littermate pairs of pigs were collected at 21 d of age for metabolomic analysis (Metabolon, Inc., Durham, NC).

**Results:**

Birth weights did not differ between littermate pairs of SG and Control pigs (*P* > 0.05). Weaning weights differed by 1.51 ± 0.19 kg (*P* < 0.001). Random forest (RF) analysis was effective at segregating the metabolome of muscle samples by growth rate, resulting in a predictive accuracy of 81% versus random segregation (50%). Decreases in sugars in the pentose phosphate pathway (PPP) in the longissimus of SG pigs were detected (*P* < 0.05). Decreases were also apparent in glycolytic intermediates (glycerol-3-phosphate and lactate) and key glycolysis-derived intermediates (glucose-6-phosphate and fructose-6-phosphate; *P* < 0.05). SG pigs had increased levels of phospholipids, lysolipids, diacylglycerols, and sphingolipids (*P* < 0.05). Pathway analysis identified a cluster of molecules associated with muscle and collagen/extracellular matrix breakdown that are increased in the SG pig (glutamate, 3-methylhistidine and hydroxylated proline moieties; *P* < 0.05). Nicotinate metabolism was altered in SG pigs, resulting in a 78% decrease in the nicotinamide adenine dinucleotide pool (*P <* 0.05).

**Conclusions:**

These metabolomic data provide the first evidence for biochemical mechanisms that should be investigated to determine if they have a potential role in the slow growth in some normal birth weight piglets that contribute to increased intra-litter variability in weaning weights and provides essential information and potential targets for the development of nutritional intervention strategies.

**Electronic supplementary material:**

The online version of this article (10.1186/s40104-018-0251-3) contains supplementary material, which is available to authorized users.

## Background

Intra-litter variability in weaning weight has increased in the past decade with selection for larger litter size [1]. A major component of this variability in weaning weight is variability in birth weight [2]. However, some normal birth weight animals grow slower than littermates and these pigs cannot be identified for an intervention until weaning, when it may be too late since pigs with poor preweaning gain do not recover and will reach mature weight later than littermates [3, 4]. Recent studies have identified markers that can identify these animals at birth [5, 6]. These normal birth weight animals may be metabolically perturbed prenatally, but it does not become apparent until postnatally, producing a slower growth rate than littermates of similar birth weight.

The metabolic differences associated with growth rate have not been identified. Intervention during the first few weeks prior to weaning may be essential to ameliorate the depressed growth rate in slow growing pigs, but requires knowledge of the metabolic mechanisms contributing to or affected by the impaired growth rate. Enhancement of the preweaning growth rate is important as weight at weaning has a positive but curvilinear effect on post-weaning growth rate [7]. The aim of this study was to interrogate biochemical profiles manifested in skeletal muscle metabolome originating from slow growing and faster growing littermates, with the aim of identifying differences in metabolic pathway utilization between slow growing pig relative to their faster growing littermates.

## Methods

### Animal procedures

Birth weights for the pigs from eight litters (Large White × Poland China × Landrace) were obtained and subsequently weights were recorded every two days until d 21 of age when animals were weaned. Body weight changes were plotted and sex matched pairs of littermate pigs (six pairs of females, two pair of males) were identified based upon a divergence in growth rate ≥ 50 g/d. These eight pairs of littermate pigs were obtained from eight different sows. Birth weights of selected pigs were within one standard deviation of the mean litter birth weight to avoid using small for gestational age pigs. Control littermates grew within one standard deviation of the mean of the litter average. Pigs selected for this study were observed to be healthy.

On d 21 of age, pairs were euthanized by intravenous pentobarbital sodium (200 mg/kg) administration between 0900 and 1100 h, while animals were in the fed state. Longissimus samples were then collected. Tissues were diced and frozen in liquid nitrogen immediately upon removal from the carcass and stored at − 80 °C. Animal handling and euthanasia procedures were approved by the USDA-ARS Institutional Animal Care and Use Committee of the Beltsville Agricultural Research Center.

### Metabolomic analysis (performed by metabolon, Inc., Durham, NC)

#### Sample preparation and analysis

Samples were prepared using the automated MicroLab STAR® system from Hamilton Co. (Reno, NV, USA). Several recovery standards were added prior to the first step in the extraction process for quality control (QC) purposes. Proteins were precipitated with methanol under vigorous shaking for 2 min (GenoGrinder 2000, SPEX SamplePrep, LLC, Metuchen, NJ, USA) followed by centrifugation to remove protein, dissociate small molecules bound to protein or trapped in the precipitated protein matrix, and to recover chemically diverse metabolites. The resulting extract was divided into five fractions: two for analysis by two separate reverse phase (RP)/ultra-performance liquid chromatography-tandem mass spectrometry (UPLC-MS/MS) methods with positive ion mode electrospray ionization (ESI), one for analysis by RP/UPLC-MS/MS with negative ion mode ESI, one for analysis by hydrophilic interaction chromatography (HILIC)/UPLC-MS/MS with negative ion mode ESI, and one sample was reserved for backup. Samples were placed briefly on a TurboVap® (Zymark, Hopkinton, MA, USA) to remove the organic solvent. The sample extracts were stored overnight under nitrogen before preparation for analysis.

#### Ultrahigh performance liquid chromatography-tandem mass spectroscopy (UPLC-MS/MS)

All methods utilized a Waters ACQUITY ultra-performance liquid chromatograph (UPLC, Waters Co., Milford, MA, USA) and a Thermo Scientific Q-Exactive high resolution/accurate mass spectrometer interfaced with a heated electrospray ionization (HESI-II) source and Orbitrap mass analyzer operated at 35,000 mass resolution (Thermo Fisher Scientific, Waltham, MA, USA). The sample extract was dried then reconstituted in solvents compatible to each of the four methods. Each reconstitution solvent contained a series of standards at fixed concentrations to ensure injection and chromatographic consistency. One aliquot was analyzed using acidic positive ion conditions, chromatographically optimized for more hydrophilic compounds. In this method, the extract was gradient eluted from a C18 column (Waters UPLC BEH C18–2.1 mm × 100 mm, 1.7 μm) using water and methanol, containing 0.05% perfluoropentanoic acid (PFPA) and 0.1% formic acid (FA). Another aliquot was also analyzed using acidic positive ion conditions; however, it was chromatographically optimized for more hydrophobic compounds. In this method, the extract was gradient eluted from the same C18 column using methanol, acetonitrile, water, 0.05% PFPA and 0.01% FA and was operated at an overall higher organic content. Another aliquot was analyzed using basic negative ion optimized conditions using a separate dedicated C18 column. The basic extracts were gradient eluted from the column using methanol and water, but with 6.5 mmol/L ammonium bicarbonate at pH 8.0. The fourth aliquot was analyzed via negative ionization following elution from a HILIC column (Waters UPLC BEH Amide 2.1 mm × 150 mm, 1.7 μm) using a gradient consisting of water and acetonitrile with 10 mmol/L ammonium formate, pH 10.8. The mass spectrometry (MS) analysis alternated between MS and data-dependent MS^n^ scans using dynamic exclusion. The scan range varied slighted between methods but covered 70–1,000 *m/z*.

#### Data extraction and compound identification

Raw data was extracted, peak-identified and QC processed using Metabolon’s hardware and software. Compounds were identified by comparison to library entries of purified standards or recurrent unknown entities. Metabolon maintains a library based on authenticated standards that contains the retention time/index (RI), mass to charge ratio (*m/z)*, and chromatographic data (including MS/MS [tandem mass spectrometry] spectral data) on all molecules present in the library. Furthermore, biochemical identifications were based on three criteria: retention index within a narrow RI window of the proposed identification, accurate mass match to the library ± 10 ppm, and the MS/MS forward and reverse scores between the experimental data and authentic standards. The MS/MS scores were based on a comparison of the ions present in the experimental spectrum to the ions present in the library spectrum. While there may be similarities between these molecules based on one of these factors, the use of all three data points can be utilized to distinguish and differentiate biochemicals. More than 3300 commercially available purified standard compounds have been acquired and registered into a proprietary database for analysis on all platforms for determination of their analytical characteristics. Additional mass spectral entries were created for structurally unnamed biochemicals, which have been identified by their recurrent nature (both chromatographic and mass spectral).

#### Curation

A variety of curation procedures were carried out to ensure that a high-quality data set was made available for statistical analysis and data interpretation. The QC and curation processes were designed to ensure accurate and consistent identification of true chemical entities, and to remove those representing system artifacts, mis-assignments, and background noise. Metabolon data analysts used proprietary visualization and interpretation software to confirm the consistency of peak identification among the various samples. Library matches for each compound were checked for each sample and corrected if necessary. Following identification, metabolite peaks were quantified using area under the curve. In cases where a compound was below detection limits for an individual animal, metabolon imputed the value with the minimum value detected for that compound to facilitate statistical analysis [8]. The imputed minimum detection values were used for statistical analysis between slow growing and control groups.

### Statistical analysis

Data were subjected to principal component analysis, hierarchal analysis and random forest analysis [9]. Paired *t*-test was used to compare data between littermates of similar birth weight.

## Results

Birth weights were similar between slow growing and control pigs (1.59 ± 0.07 versus 1.56 ± 0.06 kg, respectively; *P* > 0.05). Control piglets weighed 1.51 ± 0.19 kg more at d 21 than slow growing piglets (6.98 ± 0.28 kg versus 5.47 ± 0.22 kg; *P* < 0.001), a difference in average daily gain of 39% (74 ± 8 g/d; *P* < 0.001).

**Fig. 1:**
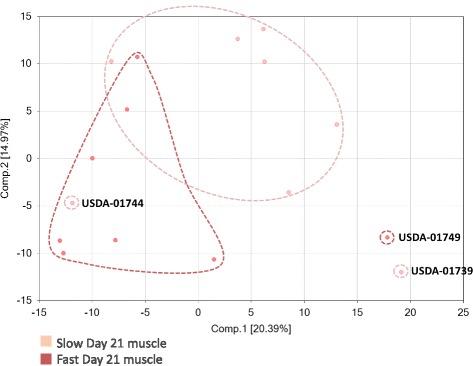
Principal component analysis of swine longissimus metabolome using orthogonal contrasts. Metabolomes that may display independent clustering by PCA are labeled according to the identification code for the longissimus metabolome of individual pigs (USDA-017##). Tissue samples collected at 21 d of age from birth weight matched pairs of pigs were used in the analysis (8 littermate pairs). Comp.1 represents component one in the principal component analysis and accounts for 20.39% of the total variance. Comp. 2 represents component 2 in the principal component analysis and accounts for 14.97% of the total variance

**Fig. 2:**
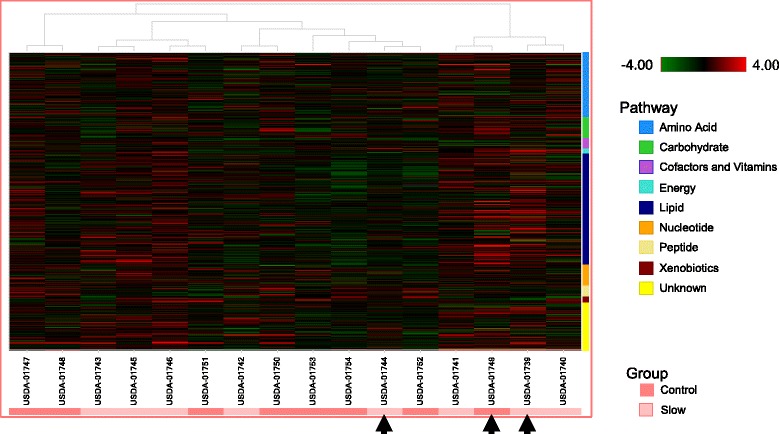
Hierarchical cluster analysis of the metabolome from the longissimus of slow and normal growing pigs. Heat map representation of metabolites that differed significantly between slow and normal muscle samples. Each block represents the abundance of one metabolite from one sample. Samples are sorted by muscle source. Cells are conditionally formatted so that the minimum value measured (or imputed) for each metabolite is green; the maximum value is red. Hierarchical cluster analysis (HCA) of the dataset revealed that the samples showed modest evidence of clustering by growth rate (X-axis pink/red bars), consistent with overlapping populations detected by PCA. Independently clustering samples identified by PCA are indicated with black arrows and show highly dispersed clustering patterns. USDA-017## represent the identification code for longissimus metabolome of individual pigs

Clustering of the muscle metabolome from distinct piglet phenotypes identified by PCA are indicated with black arrows and show highly dispersed clustering patterns (Fig. 1). Hierarchical cluster analysis (HCA) of the dataset revealed that the samples showed modest evidence of clustering by growth rate (Fig. 2; X-axis pink/red bars), consistent with overlapping populations detected by principle component analysis (PCA). Collectively, PCA and HCA indicated that there are likely metabolite differences in muscles of pigs exhibiting different growth rates.

**Fig. 3:**
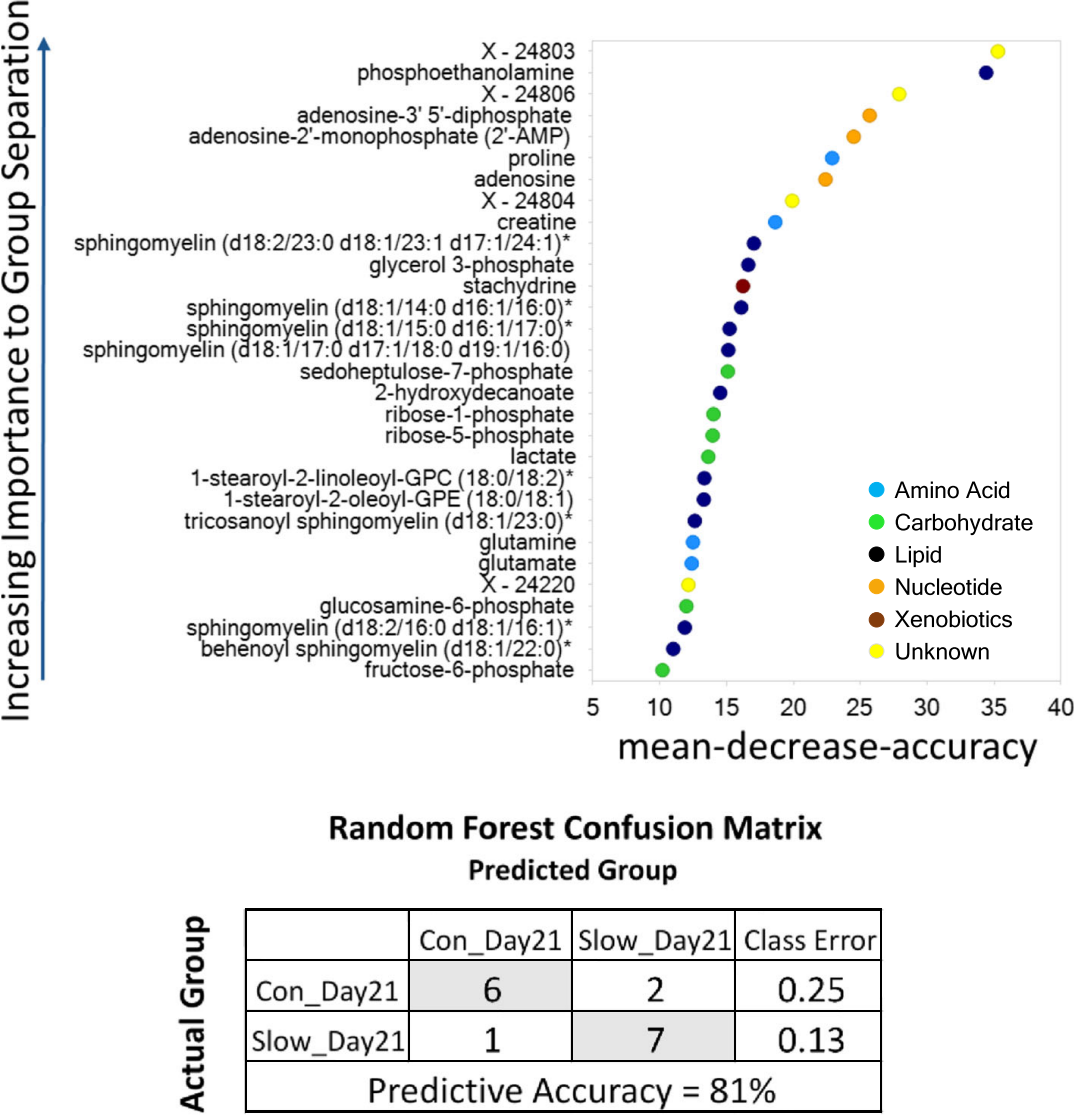
Random forest plot to document slow and control group muscle classification. Various biochemicals are shown in color dots, and mean predictive accuracy of slow and control group muscle status based on metabolomic profiling is shown in the associated table within the figure. Con_Day21 = longissimus metabolome as sampled at d 21 of age from control piglets. Slow_Day21 = longissimus metabolome as sampled at d 21 of age from slow piglets.

Random forest (RF) analysis was effective at segregating muscle samples according to growth rate, resulting in a predictive accuracy of 81% versus random segregation (50%). The results of the RF analysis and the top 30 Mean Decrease Accuracy values calculated for the comparison of all groups are shown in Fig. 3. A higher Mean Decrease Accuracy value indicates a greater group-differentiating contribution. In addition to several unnamed metabolites, predictive metabolites separating these groups included lipids (phosphoethanolamine, and several sphingomyelins), adenosine derivatives (adenosine, adenosine-3′ 5′-diphosphate, and adenosine-2′-monophosphate) as well as several carbohydrates, such as sedohepulose-7-phosphate, ribose-1-phosphate, and ribose-5-phosphate. Together, RFA was consistent with the global metabolic profiles identified by PCA and HCA.

To help identify the most significantly altered metabolites in the muscles of pig littermates exhibiting different growth rates, the dataset was sorted by *P*-value. Consistent with findings from random forest analysis, the results revealed a clear decrease in many sugars in the pentose phosphate pathway (PPP) in the longissimus of the slow growing pig. Specifically, slow growing pigs exhibited decreases in several pentose sugars and alcohols, including sedoheptulose-7-phosphate, 6-phosphogluconate, ribose-5-phosphate, and ribitol **(**Table 1). Decreases were also apparent in glycolytic intermediates (glycerol-3-phosphate and lactate) and key glycolysis-derived intermediates (glucose-6-phosphate and fructose-6-phosphate).

**Table 1:** Central carbon metabolism

Metabolite	Ratio (S21/C21)	*P*-value^a,b^
Glycolysis^c^		
Glucose-6-phosphate	0.69	0.0154
Fructose-6-phosphate	0.62	0.0116
Lactate	0.81	0.0132
Glycolysis/Glyceroneogenesis		
Glycerol-3-phosphate	0.88	0.0006
Pentose phosphate shunt		
6-Phosphogluconate	0.75	0.0222
Ribose-5-phosphate	0.49	0.0313
Sedoheptulose-7-phosphate	0.65	0.0163
Glycosylation		
Glucosamine-6-phosphate	0.74	0.0045
Mannose-6-phosphate	0.78	0.0215

^a^*n* = 8 animals per group

^b^Paired *t*-test analysis of longissimus muscle from slow growing and control littermates

^c^Metabolic pathway associated with the metabolite

Slow growing pigs exhibited significant increases in several lipid classes in the longissimus metabolome. Sorting the muscle data set by statistical significance of changes revealed that slow growing pigs displayed increased levels of several phospholipids, lysolipids, diacylglycerols, and sphingolipids (Table 2). Increases in phospholipid, lysolipid and sphingolipid moieties were the most common biochemical changes identified between longissimus samples from slow growing and control pigs. The phospholipid phosphoethanolamine demonstrated the most highly significant difference between slow growing and control longissimus (*P* = 0.0009) as shown in Table 2 and Fig. 3. The fatty acid transporter carnitine was also elevated in longissimus from slow growing pigs (Table 3).

**Table 2:** Lipid metabolism

Metabolite	Ratio	*P*-value^a,b^
Phospholipids		
Phosphoethanolamine	1.27	0.0009
1-Stearoyl-2-linoleoyl-GPC	1.16	0.0084
1-Palmitoyl-2-linoleoyl-GPE	1.18	0.0106
1-Stearoyl-2-oleoyl-GPC	1.16	0.0207
1,2-Dilinoleoyl-GPE	1.19	0.0300
1-Oleoyl-2-linoleoyl-GPE	1.19	0.0232
1-Stearoyl-2-oleoyl-GPE	1.17	0.0253
1-Linoleoyl-2-arachidonoyl-GPE	1.35	0.0405
1-Palmitoyl-2-oleoyl-GPE	1.15	0.0345
Diacylglycerols		
Palmitoyl-linoleoyl-glycerol	1.52	0.0133
Oleoyl-linoleoyl-glycerol	1.19	0.0201
Lysolipids		
1-Palmitoyl-GPE (16:0)	1.41	0.0113
1-Stearoyl-GPC (18:0)	1.39	0.0127
1-Oleoyl-GPE (18:1)	1.41	0.0172
1-Stearoyl-GPE (18:0)	1.35	0.0247
1-Oleoyl-GPI (18:1)	1.39	0.0442
Sphingolipids		
Sphingomyelin (d18:2/23:0, d18:1/23:1, d17:1/24:1)	1.28	0.0088
Sphingomyelin (d18:1/15:0, d16:1/17:0)	1.33	0.0056
Sphingomyelin (d18:1/17:0, d17:1/18:0, d19:1/16:0)	1.22	0.0114
Sphingomyelin (d18:1/14:0, d16:1/16:0)	1.19	0.0120
Behenoyl sphingomyelin (d18:1/22:0)	1.16	0.0238
Tricosanoyl sphingomyelin (d18:1/23:0)	1.20	0.0286
Sphingomyelin (d18:1/21:0, d17:1/22:0, d16:1/23:0)	1.25	0.0443
Glycosyl-N-palmitoyl-sphingosine	1.23	0.0461

*GPC* glycerophosphocholine, *GPE* glycerophosphoethanolamine

^a^*n* = 8 animals per group

^b^Paired *t*-test analysis of longissimus muscle from slow growing and control littermates

**Table 3:** Carnitine

Metabolite	Ratio (S21/C21)	*P*-value^a,b^
Carnitine	1.11	0.0034
Acetylcarnitine	1.19	0.0186
Deoxycarnitine	1.38	0.0261

^a^*n* = 8 animals per group

^b^Paired *t*-test analysis of longissimus muscle from slow growing and control littermates

Pathway analysis identified a cluster of molecules that are associated with muscle and collagen/extracellular matrix breakdown that are affected in the slow growing pig (Table 4). Muscle of slow growing pigs had higher levels of 3-methylhistidine and metabolites with hydroxylated proline groups such as prolylhydroxyproline (Pro-hydroxy-Pro) and *trans*-4-hydroxyproline. The amino acid and amino acid precursors glutamate, glutamine, proline and homocitrulline were also elevated in muscle from slow growing pigs relative to control pigs. The abundance of the creatine precursor guanidinoacetate was increased, while levels of creatine and its metabolite creatinine were reduced in longissimus from slow growing pigs.

**Table 4:** Muscle and collagen breakdown

Metabolite	Ratio (S21/C21)	*P*-value^a,b^
*Trans*-4-hydroxyproline	1.32	0.0019
3-Methylhistidine	1.49	0.0025
N-delta-acetylornithine	1.92	0.0028
Proline	1.28	0.0040
Guanidinoacetate (glycocyamine)	1.45	0.0097
Creatine	0.94	0.0111
Creatinine	0.86	0.0141
Glutamate	1.25	0.0195
Homocitrulline	1.56	0.0238
Pro-hydroxy-Pro	1.19	0.0340
Glutamine	1.30	0.0489

^a^*n* = 8 animals per group

^b^Paired *t*-test analysis of longissimus muscle from slow growing and control littermates

The skeletal muscle metabolome from slow growing pigs exhibited significant increases in several purine intermediates, such as 7-methylguanine, adenosine, guanosine, adenosine-2′-monophosphate, and adenosine-3′,5′-diphosphate (Table 5). These levels correlate inversely with nicotinamide adenine dinucleotide (NAD) and nicotinamide ribonucleotide levels observed in the longissimus; the reduction in NAD and nicotinamide ribonucleotide were proportionally greater than the increase in purine abundance in slow growing pig longissimus relative to control pigs.

**Table 5:** Purine and nicotinate metabolism

Metabolite	Ratio (S21/C21)	*P*-value^a,b^
7-Methylguanine	1.16	0.0025
Adenosine	1.56	0.0045
Guanosine	1.35	0.0056
Adenosine-2′-monophosphate	1.61	0.0071
Adenosine-3′,5′-diphosphate	1.45	0.0382
Nicotinamide adenine dinucleotide	0.22	0.0121
Nicotinamide ribonucleotide (NMN)	0.56	0.0358

^a^*n* = 8 animals per group

^b^Paired *t*-test analysis of longissimus muscle from slow growing and control littermates

## Discussion

Pigs that exhibit differential growth rates within the same litter represent a problem for the swine industry. Slow growing pigs are at a physical size disadvantage to compete with pen mates for feed, which is further exacerbated in production facilities where pigs are fed in close proximity to one another. This is problematic to pork producers due to animal well-being concerns and reduced profitability; thus, there is a great deal of interest to identify metabolic pathways affected by this growth variability to permit development of strategies to improve performance. The goal of this study is to gain insight into the metabolic alterations associated with differential growth rates among neonatal swine, with a secondary goal of identifying potential markers predictive for postnatal growth. Analysis of samples prepared from the longissimus muscle of these littermate pigs at 21 d of age exhibited evidence of moderate group clustering following PCA and HCA analysis, forming two partially overlapping populations. This result suggests that muscle tissue may harbor growth-rate specific metabolic alterations in the slow growing pig versus its littermates.

Previous research with mice overexpressing serine/threonine kinase 1 (Akt) demonstrated an increase in muscle growth that was accompanied by an increase in PPP metabolites [10]. Another study has suggested that glycolysis involving the PPP plays an essential role in platelet-derived growth factor–induced vascular smooth muscle cells proliferation by providing substrates that enhance the mitochondrial reserve capacity [11]. Higher glucose utilization along the PPP axis may also potentiate cardiac muscle stem cell outgrowth to facilitate increased growth responses [12]. Interestingly, many pathophysiological conditions in skeletal muscle are also associated with an increase in PPP enzyme active and elevated PPP metabolites [13], whereas a decrease in PPP metabolites have not been reported to affect muscle growth. However, lower glucose-6-phosphate dehydrogenase activity has been reported in skeletal muscle of intra-uterine growth retarded (IUGR) pigs relative to normal sized littermates [14]. The same study also reported lower lactate dehydrogenase (LDH) activity, suggesting a lower level of lactate production in skeletal muscle of the IUGR pig relative to littermates. However, LDH activity is sensitive to dietary restriction in the neonatal pig [15]; which may complicate the interpretation as IUGR pigs have difficulty competing with littermates to obtain adequate nutrition. In addition to these changes in carbon metabolism, decreases were apparent in the pentose sugars mannose-6-phosphate and glucosamine-6-phosphate, which could correlate with changes in protein and lipid glycosylation [16, 17].

The smaller accretion of pentose phosphate metabolites, with corresponding accretion of many lipid classes by muscle from slow growing pigs may represent a shift away from carbohydrate oxidation to elevated fatty acid metabolism and β-oxidation to fuel growth, derived from the lipid rich milk. The increase in lysolipids and diglycerides supports this hypothesis. The increase in phospholipids may suggest a shift in mitochondrial metabolism or an increase in mitochondrial membrane remodeling [18]. Recent work also indicates that phospholipids may have a role in skeletal muscle inflammation, outside of the well characterized role of inositol phospholipids [19].

Among the metabolites evaluated in this study, the phospholipid phosphoethanolamine may best serve as a potential marker for discriminating slow growing pigs from their littermates. This is supported by the random forest analysis which indicates that it is most predictive for detecting a differential growth rate among the metabolites quantified (Fig. 3). An increase in phosphoethanolamine (P-Etn) may indicate some limitation in the activity of CTP:phosphoethanolaminecytidylyltransferase (pCyt2), the rate limiting enzyme in phosphatidylethanolamine (PE) synthesis present in skeletal muscle of slow growing pigs.

Interestingly, sphingolipids may also serve as a precursor for P-Etn [20]. Since sphingolipids were also increased in the muscle of slow growing pigs, this provides more precursor for P-Etn synthesis and subsequently for the synthesis of PE. Therefore, accumulation of sphingolipids, diglycerides and P-Etn suggests a potential inhibition of pCyt2 may exist in skeletal muscle of the slow growing pig, which may result in reduced PE synthesis, a phospholipid that has possible roles in skeletal muscle growth and maintenance, mitochondrial biogenesis, oxidative capacity, and exercise performance [21].

An increase in sphingolipids (ceramides) is associated with the development of insulin resistance [22] and oxidative stress in inflammatory diseases [23] within skeletal muscle. In addition, accumulation of diglycerides has also been associated with development of insulin resistance in muscle [24]. Therefore, the higher levels of diglycerides and sphingolipids in the skeletal muscle of slow growing pigs suggest the onset of the development of an inflammatory state within the skeletal muscle in these young pigs relative to their fasting growing littermates, although this requires further studies.

Metabolism of carnitine, along with associated intermediates acetylcarnitine and deoxycarnitine, is intimately linked to fatty acid metabolism and glycerolipid processing via mitochondrial uptake of fatty acids by the carnitine shuttle [25]. Increased levels of carnitine, coupled with increased availability of phospholipids, diacylglycerols, and sphingolipids, imply that an elevated flux of lipids may occur across the mitochondrial membranes of slowly growing animals. An increased flux of phospholipids across the membrane could then contribute to mitochondrial membrane remodeling, but this requires further investigation.

The histidine metabolite 3-methylhistidine is prevalent in skeletal muscle and is used as a marker of muscle protein breakdown/turnover [26]. Pro-hydroxy-Pro and *trans*-4-hydroxyproline are commonly found in collagen/extracellular matrix and released with breakdown of these proteins [27, 28]. Increases in the levels of these amino acid derivatives could correlate with increased muscle breakdown and extracellular matrix remodeling in slow growing pigs at d 21 of age. Alterations in several other metabolites may suggest a broader metabolic context within the arginine cycle [29]. Given that slow growing pig muscle accumulates several downstream derivatives, including glutamate, proline, and homocitrulline, with concomitant decreases in creatine and creatinine, the results suggest that muscle from control pigs may better utilize of the arginine pathways to potentiate muscle growth relative to longissimus from slow growing pigs.

NAD is a coenzyme that plays a vital role in redox reactions, transferring electrons to NAD^+^ by reduction to NADH, as part of β-oxidation, glycolysis, and the TCA cycle. The observed increases in purine abundance in muscle of slow growing pigs, when coupled with the absence of significant changes in purine breakdown markers such as xanthine, xanthosine (*P* > 0.05, Additional file 1), possibly suggests that metabolic flow may be shunted away from NAD^+^ production in longissimus from slow growing pigs. Furthermore, nicotinamide metabolism is known to impact glucose, lipid, and energy metabolism through regulating the action of sirtuins, a family of a NAD-dependent deacetylases that regulate several epigenetic and signal transduction cascades through post-translational modification of histone and non-histone targets [30–32]. The large decrease in NAD abundance suggests a key metabolic difference between the slow growing pig relative to its littermate.

## Conclusions

The impaired growth rate in the slow growing piglet relative to its littermate is associated with a shift in the metabolome from central carbon metabolism to lipid oxidation and an increase in muscle and extracellular matrix protein breakdown, consistent with a more catabolic state in the slow growing pig cohort. This is paralleled by shifts in the levels of phospholipids and sphingolipids that suggest changes in mitochondrial membrane composition and perhaps fluidity, accompanied by a large decrease in NAD levels that occur in the longissimus of slow growing pigs. Whether the metabolic changes are causative of or responsive to perturbations in growth rate in young pigs cannot be ascertained in this study, but can only be associated. These data also suggest that feed intake may be reduced in the slower growing littermates relative to the control pigs, but whether this is an indirect consequence of the reduced metabolic body size resulting from impaired tissue growth and metabolism or a specific restriction/reduction in intake is unclear and requires further investigation. The present data are correlative at best but do provide the first evidence for biochemical mechanisms that should be investigated for their potential role, if any, in the slow growth in some normal birth weight piglets that contributes to increased intra-litter variability in weaning weight and provides new information and potential targets for the development of nutritional intervention strategies.

## Additional file

Additional file 1: Supplemental muscle data. (XLSX 1373 kb)

## Abbreviations

Akt: Serine/threonine kinase 1
ESI: Electrospray ionization
FA: Formic acid
HCA: Hierarchical cluster analysis
HESI-II: Heated electrospray ionization
HILIC: Hydrophilic interaction chromatography
IUGR: Intra-uterine growth retarded
LDH: Lactate dehydrogenase
MS: Mass spectrometry
MS/MS: Tandem mass spectrometry
NAD: Nicotinamide adenine dinucleotide
PCA: Principle component analysis
pCyt2 CTP: Phosphoethanolaminecytidylyltransferase
PE: Phosphatidylethanolamine
P-Etn: Phosphoethanolamine
PFPA: Perfluoropentanoic acid
PPP: Pentose phosphate pathway
Pro-hydroxy-Pro: Prolylhydroxyproline
QC: Quality control
RF: Random forest
RI: Retention time/index
RP: Reverse phase
SG: Slow growing
UPLC: Ultra-performance liquid chromatograph
UPLC-MS/MS: Ultra-performance liquid chromatography-tandem mass spectrometry
